# *In vitro* activities of amphotericin B deoxycholate and liposomal amphotericin B against 604 clinical yeast isolates

**DOI:** 10.1099/jmm.0.075507-0

**Published:** 2014-12

**Authors:** Maria Teresa Montagna, Grazia Lovero, Caterina Coretti, Osvalda De Giglio, Domenico Martinelli, Andrea Bedini, Mario Delia, Antonio Rosato, Mauro Codeluppi, Giuseppina Caggiano

**Affiliations:** 1 Department of Biomedical Science and Human Oncology, Hygiene Section, University of Bari, Bari, Italy; 2 Department of Medical and Surgical Sciences, Hygiene Section, University of Foggia, Foggia, Italy; 3 Department of Internal Medicine and Medical Specialties, Infectious Diseases Clinic, University of Modena and Reggio Emilia, Modena, Italy; 4 Department of Emergency and Organ Transplantation, Hematology Section, University of Bari, Bari, Italy; 5 Department of Pharmaceutical Chemistry, Section of Microbiology, Faculty of Pharmacy, University of Bari, Bari, Italy

## Abstract

We determined the *in vitro* antifungal activity of liposomal amphotericin B (L-AmB) against 604 clinical yeast isolates. Amphotericin B deoxycholate (D-AmB) was tested in parallel against all the isolates. Susceptibility testing was performed according to the Clinical and Laboratory Standards Institute (CLSI) M27-A3 method. Overall, L-AmB was highly active against the isolates (mean MIC, 0.42 µg ml^−1^; MIC_90_, 1 µg ml^−1^; 97.2 % of MICs were ≤1 µg ml^−1^) and comparable to D-AmB (mean MIC, 0.48 µg ml^−1^; MIC_90_, 1 µg ml^−1^; 97.3 % of MICs were ≤1 µg ml^−1^). The *in vitro* activity of D-AmB and L-AmB was correlated (*R*^2^ = 0.61; exp(*b*), 2.3; 95 % CI, 2.19–2.44, *P*<0.001). *Candida albicans* (mean MICs of D-AmB and L-AmB, 0.39 µg ml^−1^ and 0.31 µg ml^−1^, respectively) and *Candida parapsilosis* (mean MICs of D-AmB and L-AmB, 0.38 µg ml^−1^ and 0.35 µg ml^−1^, respectively) were the species most susceptible to the agents tested, while *Candida krusei* (currently named *Issatchenkia orientalis*) (mean MICs of D-AmB and L-AmB, 1.27 µg ml^−1^ and 1.13 µg ml^−1^, respectively) was the least susceptible. The excellent *in vitro* activity of L-AmB may have important implications for empirical treatment approaches and support its role in treatment of a wide range of invasive infections due to yeasts.

## Introduction

Amphotericin B deoxycholate (D-AmB), a polyene macrolide, is the longest established antifungal agent and for many decades was considered the gold standard for the treatment of invasive fungal infections. It is active against many clinically relevant yeasts (i.e. *Candida* spp. and *Cryptococcus neoformans*) and moulds, including most of *Aspergillus* spp. and *Mucorales* (Adler-Moore & Proffitt, 2002; Lacerda & Oliveira, 2013; Moen *et al.*, 2009). Acquired resistance to this agent is rare (Kanafani & Perfect, 2008). The clinical use of D-AmB is impaired by its poor aqueous solubility and its toxicity, especially nephrotoxicity (nearly 50 % of patients), and by infusion-related reactions, such as fever and chills (Dupont, 2002). As a result of these limitations, in 2009 the Infectious Diseases Society of America (IDSA) guidelines (Pappas *et al.*, 2009) introduced a significant change in the management of patients with invasive candidiasis: D-AmB, previously recommended as first-line therapy (Pappas *et al.*, 2004), is now considered as an acceptable therapy only for invasive candidiasis in non-neutropenic patients intolerant or with limited access to other antifungal agents. To attenuate its adverse effects, lipid formulations of amphotericin B [liposomal amphotericin B (L-AmB), amphotericin B lipid complex and amphotericin B colloidal dispersion] were developed (Table 1). Several studies have indicated that the three lipid-based formulations are not therapeutically equivalent. L-AmB appears to be substantially less toxic than the other two formulations in terms of nephrotoxicity and incidence of infusion-related adverse events (Cifani *et al.*, 2012; Enoch *et al.*, 2006; Saliba & Dupont, 2008; Wade *et al.*, 2013). Based on its enhanced safety and efficacy profile, the US Food and Drug Administration (www.fda.gov), the European Society of Clinical Microbiology and Infectious Diseases (ESCMID) (Ullmann *et al.*, 2012) and the European Conference on Infections in Leukaemia (ECIL; Maertens *et al.*, 2011) guidelines propose L-AmB for empiric antifungal therapy in febrile neutropenic patients. L-AmB is also recommended (AII recommendation) as therapy, with the same strength of recommendation as echinocandins, according to IDSA guidelines for neutropenic patients with candidaemia (Pappas *et al.*, 2009). Moreover, whereas the clinical activity of L-AmB has been widely studied (Cifani *et al.*, 2012; Enoch *et al.*, 2006; Saliba & Dupont, 2008; Wade *et al.*, 2013), there is not an extensive literature (Anaissie *et al.*, 1991; Carrillo-Muñoz *et al.*, 1999; Jessup *et al.*, 2000; Lass-Flörl *et al.*, 2008) concerning the *in vitro* susceptibility of this agent against yeasts.

**Table 1.** Structural features of the different formulations of amphotericin B

Formulation	Chemical name	Particle shape
Amphotericin B deoxycholate	Sodium deoxycholate-amphotericin B	Colloidal dispersion
Amphotericin B lipid complex	DMPC-DMPG-amphotericin B	Ribbon-like structure
Liposomal amphotericin B	HSPC-cholesterol-DSPG-amphotericin B	Small uniform spherical lipid vesicles
Amphotericin B colloidal dispersion	Sodium cholesteryl sulfate-amphotericin B	Disc-like structure

DMPC, dimyristoylphosphatidylcholine; DMPG, dimyristoylphosphatidylglycerol; DSPG, distearoylphosphatidylglycerol; HSPC, hydrogenated soy phosphatidylcholine.

The aim of this study was to investigate the *in vitro* susceptibility to L-AmB compared with D-AmB of clinically relevant yeasts obtained from critically ill and haematological patients with bloodstream infection. Comparisons between susceptibility testing results were undertaken in order to better understand the activity profile of L-AmB compared with D-AmB.

## Methods

### 

#### Clinical isolates.

Between January 1998 and December 2012, a total of 604 yeast isolates were collected from patients with bloodstream infection admitted to two Italian hospitals. For this study, we did not use any additional data or samples other than those obtained through routine laboratory collection. Therefore, neither ethical approval nor patient consent was considered necessary. The following yeast isolates were collected (currently valid names are shown in parentheses, Schmalreck *et al.*, 2014): *Candida albicans* (*n* = 251), *Candida parapsilosis* (*n* = 224), *Candida tropicalis* (*n* = 46), *Candida glabrata* (*n* = 37), *Candida guilliermondii* (*Meyerozyma guilliermondii*, *n* = 15), *Candida krusei* (*Issatchenkia orientalis*, *n* = 11), *Candida lusitaniae* (*Clavispora lusitaniae*, *n* = 8), *Candida norvegensis* (*Pichia norvegensis*, *n* = 6), *Candida dubliniensis* (*n* = 2), *Candida kefyr* (*Kluyveromyces marxianus*, *n* = 2), *Candida intermedia* (*n* = 1) and *Candida pelliculosa* (*Wickerhamomyces anomalus*, *n* = 1). The isolates were identified using standard procedures [i.e. morphology on cornmeal agar plates, germ-tube production in serum and biochemical analysis using the ID32C and VITEK-2 System (bioMérieux)]. Isolates were frozen at −80 °C until analysis. Prior to being tested, each isolate was subcultured on Sabouraud dextrose agar plates (bioMérieux) to ensure purity, viability and optimal growth characteristics.

#### Susceptibility testing.

D-AmB (Sigma-Aldrich) and L-AmB (AmBisome; Gilead Sciences) were obtained as standard powders. Broth microdilution (BMD) testing was performed in accordance with the Clinical and Laboratory Standards Institute (CLSI) method M27-A3 (CLSI, 2008). Briefly, BMD panels containing serial twofold dilutions of each antifungal agent in RPMI 1640 medium (Sigma) buffered to pH 7.0 with MOPS (Sigma), frozen in 96-well plates at −80 °C for no more than 3 months, were thawed and inoculated with an organism suspension adjusted to attain a final inoculum concentration of 1.5×10^3^±1.0×10^3^ cells ml^−1^. The final range of drug concentrations tested was 0.03–16 µg ml^−1^. The minimum inhibitory concentration (MIC) values were visually determined, after 48 h of incubation at 35 °C, as the concentration that inhibited 100 % of fungal growth. The quality control isolates *Candida krusei* ATCC 6258 and *Candida parapsilosis* ATCC 22019 listed in CLSI (2008) were tested.

#### Analysis of results.

CLSI has not determined breakpoints for amphotericin B. In order to perform a comparison in this study, the isolates inhibited by D-AmB or L-AmB at ≤1 µg ml^−1^ were considered susceptible, as detailed in a previous study (Diekema *et al.*, 2009). ‘Resistant’ isolates were defined as isolates with MICs >1 µg ml^−1^. MIC data are presented as the range, mean, MIC_50_ (MIC causing inhibition of 50 % of isolates) and MIC_90_ (MIC causing inhibition of 90 % of isolates) and for each species. MIC_50_ and MIC_90_ values were calculated for those species with 10 or more isolates.

To analyse the correlation between the two drugs, we built a simple regression model computing *R*^2^ and exp(*b*) values with 95 % confidential interval (95 % CI). Moreover, to assess the differences in MICs among the species for each drug, the Kruskal–Wallis test was performed. The level of significance was set at a *P*-value <0.05. All statistical analyses were carried out using stata MP 11.2 for Mac Os X. Data were presented graphically using Excel.

## Results and Discussion

Table 2 summarizes the *in vitro* susceptibilities to the two different formulations of amphotericin B of 604 yeast isolates. Despite the differences in the structure, the spectra of activity of D-AmB and L-AmB are comparable. As shown by other authors (Anaissie *et al.*, 1991; Carrillo-Muñoz *et al.*, 1999; Jessup *et al.*, 2000; Lass-Flörl *et al.*, 2008), the two formulations demonstrate excellent potency and spectra: only 15 (2.5 %) and 16 (2.6 %) isolates had MIC levels that indicated resistance to D-AmB and L-AmB, respectively. D-AmB and L-AmB were statistically different with respect to yeast species (*P* = 0.001). *Candida albicans* (mean MICs of D-AmB and L-AmB, 0.39 and 0.31 µg ml^−1^, respectively) and *Candida* *parapsilosis* (mean MICs of D-AmB and L-AmB, 0.38 and 0.35 µg ml^−1^, respectively) appeared as the species most susceptible to the agents tested, in accordance with data from previous studies (Anaissie *et al.*, 1991; Carrillo-Muñoz *et al.*, 1999; Jessup *et al.*, 2000; Lass-Flörl *et al.*, 2008). Moreover, although *Candida lusitaniae* (*Clavispora lusitaniae*) is known to be intrinsically less susceptible to polyenes (Pappas *et al.*, 2004), none of our strains exhibited MIC >1 µg ml^−1^ for either agent. Other authors (Lass-Flörl *et al.*, 2008) have also reported a high susceptibility rate (98 %) by the EUCAST method [Subcommittee on Antifungal Susceptibility Testing (AFST) of the ESCMID European Committee for Antimicrobial Susceptibility Testing (EUCAST), 2008].

**Table 2.** *In vitro* susceptibilities of 604 yeast clinical isolates to deoxycholate (D-AmB) and liposomal (L-AmB) amphotericin B

Isolates	MIC (µg ml^−1^)				No. of isolates with MIC (µg ml^−1^) of:							No. resistant isolates (%)
	Range	50 %*	90 %*	Mean	0.06	0.125	0.25	0.5	1	2	4
*Candida albicans* (*n* = 251)												
D-AmB	0.06–2	0.25	1	0.39	6	39	95	82	28	1	–	1 (0.4)
L-AmB	0.06–1	0.25	0.5	0.31	8	65	108	54	16	–	–	0 (0)
*Candida parapsilosis* (*n* = 224)												
D-AmB	0.06–2	0.25	1	0.38	2	47	104	41	28	2	–	2 (0.9)
L-AmB	0.06–2	0.25	0.5	0.35	7	59	86	50	20	2	–	2 (0.9)
*Candida tropicalis* (*n* = 46)												
D-AmB	0.25–2	1	1	0.83	–	–	6	15	21	4	–	4 (8.7)
L-AmB	0.25–2	0.5	1	0.73	–	–	9	19	14	4	–	4 (8.7)
*Candida glabrata* (*n* = 37)												
D-AmB	0.25–2	0.5	1	0.75	–	–	7	16	10	4	–	4 (10.8)
L-AmB	0.125–2	0.5	1	0.72	–	1	6	18	8	4	–	4 (10.8)
*Candida gulliermondii* (*n* = 15) (*Meyerozyma guilliermondii*)												
D-AmB	0.25–2	0.5	1	0.62	–	–	7	3	4	1	–	1 (6.7)
L-AmB	0.06–2	0.25	1	0.58	1	1	6	4	1	2	–	2 (13.3)
*Candida krusei* (*n* = 11) (*Issatchenkia orientalis*)												
D-AmB	0.5–4	1	2	1.27	–	–	–	2	7	1	1	2 (25.0)
L-AmB	0.5–2	1	2	1.13	–	–	–	3	5	3	–	3 (37.5)
*Candida lusitaniae* (*n* = 8) (*Clavispora lusitaniae*)												
D-AmB	0.125–1	na	na	0.42	–	1	3	3	1	–	–	0 (0)
L-AmB	0.125–1	na	na	0.42	–	1	3	3	1	–	–	0 (0)
*Candida norvegensis* (*n* = 6) (*Pichia norvegensis*)												
D-AmB	0.125–2	na	na	0.67	–	2	1	1	1	1	–	1 (16.6)
L-AmB	0.125–2	na	na	0.61	–	3	1	–	1	1	–	1 (16.7)
Other yeasts (*n* = 6)†												
D-AmB	0.125–1	na	na	0.52	–	1	2	1	2	–	–	0 (0)
L-AmB	0.125–1	na	na	0.56	–	1	2	–	3	–	–	0 (0)

The broth microdilution test according to CLSI M27-A3 was used with 48 h incubation. na, Not applicable.

* MICs encompassing 50 and 90 % of isolates tested, respectively.

† Includes: *Candida dubliniensis* and *Candida kefyr* (*Kluyveromyces marxianus*), two isolates each; *Candida intermedia* and *Candida pelliculosa* (*Wickerhamomyces anomalus*), one isolate each.

A decrease in the activity of D-AmB and L-AmB was noted among isolates of *Candida* *glabrata* (mean MICs of D-AmB and L-AmB, 0.75 and 0.72 µg ml^−1^, respectively), as already reported by other investigators (Carrillo-Muñoz *et al.*, 1999; González *et al.*, 2008; Hull *et al.*, 2012a; Pfaller *et al.*, 2002). Recently published data (Hull *et al.*, 2012b) demonstrate that missense mutation in ERG11 enables *Candida* *glabrata* to circumvent the inhibitory action of polyenes and azoles.

Regarding *Candida krusei* (*Issatchenkia orientalis*) susceptibility, MICs of D-AmB and L-AmB were found to be the highest (mean MICs of D-AmB and L-AmB, 1.27 and 1.13 µg ml^−1^, respectively) as in other studies (Kiraz & Oz, 2011; Lass-Flörl *et al.*, 2008; Pfaller *et al.*, 2002; Ranque *et al.*, 2012). However, the molecular mechanisms influencing the susceptibility of this species to polyenes are poorly understood.

**Fig. 1.**
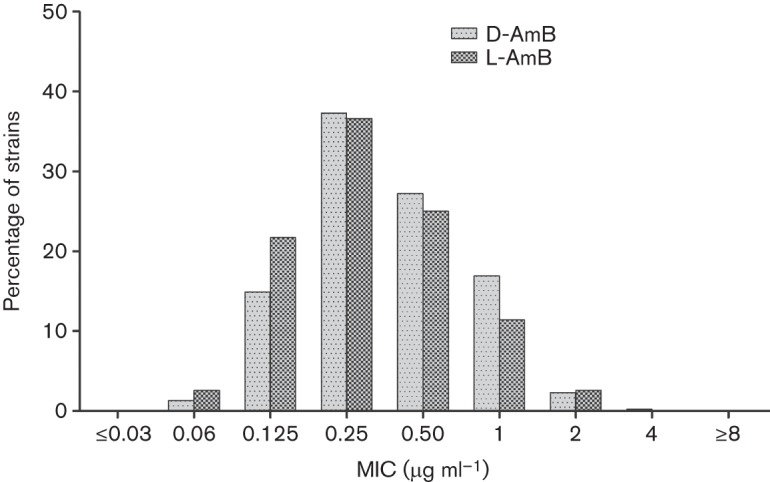
Distribution of deoxycholate and liposomal amphotericin B minimum inhibitory concentrations (MIC) for 604 clinical yeast isolates.

The overall distribution of D-AmB and L-AmB MIC values is shown in Fig. 1. The mean MIC was 0.48 and 0.42 µg ml^−1^ for D-AmB and L-AmB, respectively; MIC_50_ and MIC_90_ of D-AmB were identical to those of L-AmB (MIC_50_, 0.25 µg ml^−1^; MIC_90_, 1 µg ml^−1^). The *in vitro* activity of D-AmB against all isolates was correlated with that for L-AmB: a scatterplot of D-AmB and L-AmB MICs (Fig. S1, available in the online Supplementary Material) showed a high level of correlation (*R*^2^ = 0.61; exp(*b*) = 2.3; 95 % CI, 2.19–2.44; *P*<0.001). These results are in line with those of earlier investigators (Anaissie *et al.*, 1991; Carrillo-Muñoz *et al.*, 1999): L-AmB and D-AmB have been shown to have comparable *in vitro* activity, suggesting that the process of incorporation of amphotericin B into the liposome bilayer of L-AmB does not have any inhibitory effect on its MIC *in vitro* and does not alter its spectrum of antifungal activity (Adler-Moore & Proffitt, 2002; Lacerda & Oliveira, 2013).

In conclusion, we have performed a head-to-head challenge of D-AmB and L-AmB against a large collection of clinical yeast isolates using the CLSI M27-A3 BMD method. The results of this study demonstrate high levels of inhibitory activity of L-AmB, though a reduced susceptibility was detected for *Candida glabrata* and *Candida krusei* (*Issatchenkia orientalis*). In addition, we also found a strong correlation between the *in vitro* antifungal activities of D-AmB and L-AmB. For this reason, we can assume that if resistance to one of the two agents emerges, it is reasonable to assume that the other agent will also show comparable results, although confirmation of this finding will need additional investigation. Based on our *in vitro* results and on comparative data from well-controlled trials and extensive clinical experience (Lacerda & Oliveira, 2013; Miceli & Chandrasekar, 2012; Moen *et al.*, 2009) we conclude that, despite the availability of expanded-spectrum azoles and echinocandins, L-AmB remains a first-line drug choice for empirical therapy in patients with febrile neutropenia and it is also an option for the treatment of many patients with candidaemia. Constant surveillance is essential to monitor the activity of L-AmB against clinical yeast isolates in order to detect isolates with reduced susceptibility, thereby supporting the most appropriate choice of early antifungal treatment towards a better prognosis (Morrell *et al.*, 2005; Taur *et al.*, 2010).
